# Which headache disorders can be diagnosed concurrently? An analysis of ICHD3 criteria using prime encoding system

**DOI:** 10.3389/fneur.2023.1221209

**Published:** 2023-08-21

**Authors:** Pengfei Zhang

**Affiliations:** Department of Neurology, Rutgers Robert University Medical School, New Brunswick, NJ, United States

**Keywords:** headaches classification, International Classification of Headache Disorders, 3rd edition, headache diagnosis, number theory in medicine, automated diagnosis

## Abstract

**Introduction:**

Real-life headache presentations may fit more than one ICHD3 diagnosis. This project seeks to exhaustively list all logically consistent “co-diagnoses” according to the ICHD3 criteria. We limited our project to cases of two concurrent diagnoses.

**Methods:**

We included the criteria for “Migraine” (1.1, 1.2, 1.3), “Tension-type headache” (2.1, 2.2, 2.3, 2.4), “Trigeminal autonomic cephalalgias” (3.1, 3.2, 3.3, 3.4, 3.5), and “Other primary headache disorders.” We also excluded “probable” diagnosis criteria. Each characteristic in the above criteria is assigned a unique prime number. We then encoded each ICHD3 criteria into integers through multiplication in a list format; we called these criteria representations. “Codiagnoses representations” were generated by multiplying all possible pairings of criteria representations. We then manually encoded a list of logically inconsistent characteristics through multiplication. All co-diagnoses representations divisible by any inconsistency representations were filtered out, generating a list of co-diagnoses representations that were logically consistent. This list was then translated back into ICHD3 diagnoses.

**Results:**

We used a total of 103 prime numbers to encode 578 ICHD3 criteria. Once illogical characteristics were excluded, we obtained 145 dual diagnoses. Of the dual diagnoses, two contained intersecting characteristics due to subset relationships, 14 contained intersecting characteristics without subset relationships, and 129 contained dual diagnoses as a result of non-intersecting characteristics.

**Conclusion:**

Analysis of dual diagnosis in headaches offers insight into “loopholes” in the ICHD3 as well as a potential explanation for the source of a number of controversies regarding headache disorders. The existence of dual diagnoses and their identification may carry implications for future developments and testing of machine-learning diagnostic algorithms for headaches.

## Introduction

In clinical practice, patients’ headache profiles may satisfy more than one ICHD3 criteria. These “co-diagnoses” can be a source of diagnostic challenges and uncertainties. Consider the cases where headache differential diagnosis lies between cluster headache and paroxysmal hemicrania and when an indomethacin trial cannot be attempted. In these cases, management may be challenging (1, 2). A similar diagnostic dilemma exists between cluster headaches and migraines with aura: should we consider cluster headache cases with aura symptoms a special subtype of cluster headache or a subtype of migraine with aura? Or do these cases necessarily imply the co-existence of two separate headache disorders? (see de Coo et al., Rozen vs. Peng et al.) (3–6). Finally, in the cases of intractable headaches with an identifiable date of onset and lack of photo-phobia, phono-phobia, or nausea: should we consider these new daily persistent headaches or chronic tension-type headaches? (Lobo et al. vs. Nierenberg et al.) (7, 8). The reader may have ready answers for each of the scenarios above; however, one has to agree that those opinions are not likely shared among our colleagues. It is not the goal of this article to weigh in on each of these issues, but rather to identify the conditions of possibilities that give rise to their existence.

We can reformulate these dilemmas as the following: In the ICHD3 criteria, what are the headache disorders that can be diagnosed without logical contradictions? Or equivalently: Which headache presentations require more than one ICHD3 diagnosis to fully account for the clinical description? We call these sorts of questions “co-diagnosis” problems. (When co-diagnosis is restricted to two diagnoses, we use the term “dual diagnosis.”) The answer to the co-diagnosis problem is fundamental to headache classification as a scientific endeavor—it forces us to examine the limitation of our classification project and asks when the current paradigm is unable to assign unique diseases with a unique diagnosis.

We propose that the solution to the co-diagnosis problem can be obtained by a novel technique of automated diagnosis through prime number representation—this technique converts ICHD3 criteria into numerical values and as a result, allows the classification to be manipulated mathematically. This method was first presented at the American Headache Society’s 2022 Denver Scientific Meeting and is described in greater detail in phase 1 of the Methods section (9). We will demonstrate that by encoding ICHD3 as numerical data it is possible to enumerate all possible dual diagnoses for primary headache disorders in ICHD3.

## Methods

There are two phases to this study. Since our technique is novel, the first phase will provide an intuitive description and rationale of our methods. Phase 2 then presents an implementation for calculating ICHD3 dual diagnoses of primary headache disorders. Finally, we analyzed the result based on a paradigm of “intersecting characteristics” outlined below.

### Phase 1: automated diagnosis method’s theoretical construction

We will first demonstrate that any ICHD3 criteria can be readily translated into numerical data through prime number representations. Let us consider as a motivating example the migraine criteria taken from ICHD3 below. [The demonstration here is an adaptation of a more formal mathematical treatment that is available in Supplementary material (10)].

ICHD3 Criteria for Migraine without aura:

- At least five attacks fulfilling criteria B–D.
- Headache attacks lasting 4–72 h (when untreated or unsuccessfully treated).
- Headache has at least two of the following four characteristics:
  1. unilateral location
  2. pulsating quality
  3. moderate or severe pain intensity
  4. aggravation by or causing avoidance of routine physical activity (e.g., walking or climbing stairs)
- During headache at least one of the following:
  1. nausea and/or vomiting
  2. photo-phobia and phono-phobia
- Not better accounted for by another ICHD-3d diagnosis.

Notice that each of the above statements is either true or false based on specific conditions. For example, if a patient’s headache attacks last 4 to 72 h, then statement B is true. The conglomerate of all the components of the criteria—i.e. statements A to E—allows the user of ICHD3 to determine if a diagnosis is true or false. For this article, we call each of the conditions in a statement a “characteristic.” For example, in the statement D2: “photo-phobia” and “phono-phobia” are characteristics. According to D2, both characteristics need to be true in order for statement D2 to be true.

Viewed this way, ICHD3 criteria are propositional statements in disguise. A propositional statement, or simply a proposition, is a declarative sentence that is either true or false, but not both (11). When combining multiple propositional statements, two basic “operators” allow for calculation/derivation of the resultant statement’s veracity: the AND operator—also called conjunction and conventionally denoted by ∧ —and the OR operator—also called disjunction and denoted by ∨. When an AND operator is applied to two statements, the result is true only if both statements are true; when an OR operator is applied to two statements, the result is true as long as one of the two statements is true. (For a more thorough introduction to propositional logic, we refer the readers to *A Logical Introduction to Proof* by Daniel Cunningham.)

Since all criteria are logical statements in disguise, one can translate the example of migraine without aura above into the following propositional logic statement: (We use the alphanumeric designation of the criteria as short-hand for each characteristic.)

$$A\wedge B\wedge \left[\begin{array}{l} \left(C1\wedge C2\right)\vee \left(C1\wedge C3\right)\vee \left(C1\wedge C4\right)\\ \vee \left(C2\wedge C3\right)\vee \left(C2\wedge C4\right)\vee \left(C3\wedge C4\right) \end{array}\right]\wedge \left(D1\vee D2\right)\wedge E$$

Now any logical statement can be translated into its disjunctive normal form (the disjunctive normal is simply a series of logic AND statements which are connected by OR) (12). Therefore one can translate the above into the following equivalent logical statement:

$$\begin{array}{l} \left(A\wedge B\wedge C1\wedge C2\wedge D1\wedge E\right)\vee \left(A\wedge B\wedge C1\wedge C2\wedge D2\wedge E\right)\\ \vee \left(A\wedge B\wedge C1\wedge C3\wedge D1\wedge E\right)\ldots \end{array}$$

Given any criteria in disjunctive normal form, we propose the following algorithm:

Step 1: Each characteristic in the ICHD3 is assigned a unique prime number (Table 1). Negations crucial to diagnostic criteria are also assigned a prime number.
Step 2: If AND is used between two characteristics, then the corresponding prime numbers for those two characteristics are multiplied together. These are called encodings.
Step 3: All encodings that are separated by OR in the disjunctive normal form are then combined in a list.
Step 4: We do not encode the ubiquitous criteria “Not better accounted for by another ICHD-3 diagnosis,” given that it is recursively referring to the totality of ICHD3 encodings, creating a logical impasse.

**Table 1:** Prime number assignments for headache characteristics.

2	1 min to 24 h with severe intensity
3	1 to 14 days per month
5	1 to 600 s
7	15 min up to 4 h after waking
11	15 to 180 min
13	1–6 cm in diameter
17	2 to 30 min
19	30 min to 7 days in duration
23	4 to 72 h
29	Abrupt explosive intensity just before or with orgasm
31	Aggravated by physical activity
37	At least one aura symptom is positive
41	At least one aura symptom is unilateral
43	At least one aura symptom spreads gradually over 5 min
47	Between 1 s to 2 h
53	Bilateral location
59	Brainstem aura
61	Brought on by cold stimuli
67	Brought on by exercise
71	Brought on by sex
73	Brought on within 1 h of compression
79	Brought on within 1 h of traction
83	Clearly remembered onset
89	Conjunctival injection
97	Constant
101	Developing only during sleep and causing wakening
103	Each individual aura symptom lasts 5–60 min
107	Every other day to 8 per day
109	Eyelid edema
113	Fixed in size and shape
127	Forehead and facial sweating
131	Fully reversible
137	Greater than 1 per day
139	Greater than 15 days per month
149	Greater than 2 episodes
151	Greater than 20 episodes
157	Greater than 5 episodes
163	Greater than 5 min
167	Greater than 5 per day
173	Greater than 8 days per month
179	Hours to days
181	Increasing in intensity with increasing sexual excitement
191	Indomethacin responsive
193	Irregular frequency
197	Lacrimation
199	Less than 12 days per year
211	Less than 48 h
223	Max within 1 min
227	Maximal at site of compression
229	Maximal at site of traction
233	Mild to moderate pain
239	Miosis
241	Moderate to severe
251	More than 1 episode per day
257	More than 10 episodes
263	More than 3 months
269	More than 10 days per month
271	Motor aura
277	Nasal congestion
281	Nausea/vomiting
283	No conjunctival injection
293	No eyelid edema
307	No forehead and facial sweating
311	No lacrimation
313	No miosis
317	No nasal congestion
331	No nausea/vomiting
337	No orbital or supraorbital or temporal pain
347	No phonophobia
349	No photophobia
353	No ptosis
359	No restless
367	No rhinorrhea
373	Nonpulsating
379	Not aggrevated by activity
383	Orbital or supraorbital or temporal pain
389	Phonophobia
397	Photophobia
401	Provoke by cough
409	Provoke by valsalva
419	Ptosis
421	Pulsating
431	Relieve by triptan or ergot
433	Resolve within 1 h after removal of compression
439	Resolve within 1 h after removal of traction
443	Resolve within 30 min after removal of cold
449	Restless
457	Retinal aura
461	Rhinorrhea
463	Round or elliptical
467	Sensory aura
479	Severe
487	Sharply contoured
491	Single or series of stabs
499	Speech and/or language aura
503	Sudden
509	The aura is accompanied or followed within 60 min by headache
521	Two or more aura symptoms occur in succession
523	Unilateral
541	Unremitting within 24 h
547	Up to 72 h with mild intensity
557	Up to few seconds
563	Visual aura

For example, in migraine without aura the first conjunction in the distinctive normative form, when excluding criteria E, is (A∧B∧C1∧C2∧D1) . This is therefore encoding as:

$$157^{*}23^{*}523^{*}421^{*}281=223417708453$$

Repeating this procedure for the totality of migraine without aura criteria yields:

[7580756461,13242732241,16451185183,102951563551,127894697713, *223417708453* ,319149 8470081,3964735629103,4166259653173,6925948962043,7277988854713,9041302068919,538 43667737173,56580493999543,70288832213209,122786715194029,1669153699852363,17539 95313985833,2178953798609479,3806388171014899,29591598361760989,9173395492145906 59].

In other words, this algorithm ensures that each numerical identification—encoding—uniquely describes a class of disease presentation sharing similar characteristics. For example, the encoding 223417708453 describes situations that are true for characteristics 157, 23, 523, 421, and 281. Notice that in a circumstance where an arbitrary additional characteristic, let us call it *x,* is true in addition to 157, 23, 523, 421, 281, then the new number x*157*23*523*421*281 will divide our encoding of 223417708453 without remainder. We will see how this property can be exploited to automate the diagnosis below.

Now a patient’s headache profile, thought of as a collection of headache characteristics, can be expressed using only logical conjunction. Therefore, a patient profile can be expressed as an integer. For example, a patient who has the phenotype of the following characteristic: five headaches, each lasting 4 to 72 h, unilateral, pulsating, with nausea, and photophobia can be represented as 157*23*523*421*281*397 = 8869683025841.

We now observe that a patient profile, represented/encoded as a number, must divide without remainder at least one number in its corresponding diagnosis’ encoding, assuming that a diagnosis exists. This observation forms the basis for automated diagnosis. For example, the encoding of a migraine without aura patient’s profile (such as 88696830255841 above) must be divided by at least one number in the migraine without aura encodings (in this case, 223417708453) without remainder.

How this method works can be understood intuitively: Consider a patient profile satisfying a migraine diagnosis. Then this patient’s profile, when translated into a propositional statement, must satisfy one of the many conjunctions in the migraine criteria’s disjunctive normal forms (Since that is the implication of the OR operator). Encoding both the patient’s profile and the conjunction that it satisfies using our algorithm implies that both must share at minimum a collection of prime numbers. Since both sets of prime numbers are “bundled” together by multiplication in the algorithm, one of the two encodings must divide the other without remainder. The bigger of the two encodings must be the patient profile since it has more “variables” to encode than those in the criteria. Therefore, the patient profile’s encoding must be divisible by the encoding of one of the conjunctions in the disjunctive normal form. Notice we use prime numbers as building blocks for encoding since they have the property of not being able to be divided by each other.

A caveat: although theoretically possible, in practice logical conflicts cannot exist within a patient’s clinical profile unless there exists more than one headache diagnosis—for example, it makes no sense to be both photophobic and not photophobic at the same time in either the criteria or user encoding. We will apply this observation directly below for a dual-diagnosis problem below.

Armed with the prime number automated diagnosis above, we can now tackle the uniqueness problem as follows: To obtain all possible combinations of dual diagnoses, we multiplied all the encodings of each ICHD3 diagnosis by those from another. This list, let us call it M, represents patient profiles that satisfy two ICHD3 diagnoses concurrently. Not every pairing of two ICHD3 encodings is possible as some of these pairings would contain characteristics that are contradictory. This contradiction is manifested by the co-occurrence of two logically contradictory characteristics in the same patient profile—if A and B are logically contradictory characteristics, then its encoding is simply the prime representation of A multiplied by the prime representation of B. Therefore, to obtain a logically consistent list of dual diagnoses, we simply eliminate from the list M those that are divisible by A*B.

### Phase 2: implementation of automated diagnosis for the purpose of extracting dual diagnoses

Due to the limitations in computational power, we only included ICHD3 primary diagnosis up to two layers deep in terms of ICHD3 classification. ICHD3 criteria for “Migraine” (1.1, 1.2, 1.3), “Tension-type headache” (2.1, 2.2, 2.3, 2.4), “Trigeminal autonomic cephalalgias” (3.1, 3.2, 3.3, 3.4, 3.5), and “Other primary headache disorders” are included in this study. We excluded “Complications of migraine”(1.5) and “Episodic syndrome that may be associated with migraine” (1.6) since these diagnoses require diagnoses of migraine as the first assumption. We also excluded “probable” diagnosis criteria.

We then encoded all included criteria into their numerical encodings (Table 2). All possible take-two combinations encodings in Table 2 are multiplied together. We called this list M’ which represents the list M that was described in the last section.

**Table 2:** Prime number encodings for selected primary headache disorders.

**Migraine without aura:**
7580756461,13242732241,16451185183,102951563551,127894697713,223417708453,3191498470081,3964735629103,4166259653173,6925948962043,7277988854713,9041302068919,53843667737173,56580493999543,70288832213209,122786715194029,1669153699852363,1753995313985833,2178953798609479,3806388171014899,29591598361760989,917339549214590659.
**New daily persistent headache:**
1145520433.
**Primary exercise headache:**
2106413.
**Primary thunderclap headache:**
17411171.
**Cold-stimulus headache:**
4026427.
**External compression headache:**
1069111207.
**External traction headache:**
1183350401.
**Cluster headache:**
1577982193416431,1932584933510011,2251727399594233,3492836989921763,4237502744118281,4911247950296083,7428927404960501,7960831515100871,8173593159157019.
**Paroxysmal Hemicrania:**
699200435231955421,856324128542507201,997735452522003803,1547668379108935033,1877628135061093771,2176163152351142153,3291741374856059791,3527426914821887461,3621701130808218529.
**Short lasting unilateral neuralgiform headaches:**
444401003390335,544266397410635,634145252028905,983674131099955,1193391458542585,1383135707181155,2092180004725285,2301897332167915.
**Hemicrania continua:**
54659995058027,66943140014887,77997970476061,120988977825071,146783582234477,170121557652511,257331886846217,275756604281507,283126491255623.
**Migraine with aura:**
75121389451,179941932871,188719588133,209121705769,345049093919,581872457273,594604896163,635348700611,716836309507,826512945899,866830650577,889227610013,910191718697,932604566599,954591314731,960542072261,1033426681907,1057790375783,1393787513933,1424286146623,1461777148759,1493763519629,1521881771231,1596119906413,1619807110787,1655251467697,1717073020447,1768673409809,1800832679981,1995517294033,2233913264179,2286579195751,2475417400847,2533776946643,2596169469181,2657375822089,4084418344297,4180711114693,4283658263531,4384648242239,4746756454183,4858664268427,6887746063999,7038462608069,7050129075331,7204398858161,7223733676877,7381802247487,7394037810713,7520755549093,7555832948803,7698062163217,7887621673439,8004677858161,8073577390691,8179834922891,8193393249709,8372679754079,8485341431141,8685388773329,8740337530027,8899260525343,8946396568063,9109066274467,9861342744299,10093830196031,10260855840551,10502762068619,11299697190653,11370137553043,11638195805767,11924778409289,12205912674341,12521286076129,13132080518867,17303362063217,17681991430027,17711299872173,18098855668063,18893605403819,19173995799781,19339034214911,19593558071111,19626034993489,20055488713259,20109312668063,20549341391653,20583402554147,20936157339367,21033805235857,21316833351403,21429740616523,21819391308607,21957433307141,22475093817329,23621355875879,24178244423051,24773617138117,25357670980273,31455913801007,51901998960457,57513025875101,60318539332423,87524773154719,89439976068389,95568625392133,96986910793067,99109162670377,101717979612241,103943755971371,105900368677769,107825924039621,111066240320587,119482780692553,125311209019019,144483943052083,243650044187461,248981555001191,266042389605127,300164058812999.
**Chronic migraine:**
47943440512446421,83751819318423001,104043233975143063,651102853410965911,808852238322886393,1412974242049523533,20184188455739943241,20663622860864407451,25074419388009478183,26348930066400847453,36097034126240313431,43802201503535229523,44842633843286660153,46028628871181563393,57180458193890635759,74589881356425382327,178668785574693357667,187384336090532058041,207642102154373361613,280625329820126307641,340526792333935171453,342607760128665739163,348615314717164035383,357835469611443767023,444531949184762684449,577755521692989825821,590397874465265314351,608991898323344642723,630853403336546877647,711764461079110004239,776547512891224440469,820665099843083049623,860697543737867588629,882936037451639990801,903751818295293585869,926006088059037063523,947837272846283516887,953745926844664084697,1026114854335689719039,1050306167208043897091,1383926017078557024641,1414208862091216915771,1451434603277511025843,1483194660242007984833,1511113966131728567387,1584826842528398253601,1608346452280485190799,1643540028916819658869,1704924174212751870619,1756159474153089956693,1788091207101178791137,1981398364625630552341,2218107606280949245183,2270400909375981447427,2457903023176187001419,2515849656335547009311,2577800731623805879537,2638574029815329790253,4055519765243973517069,4151131233186856979161,4253349997694899054487,4353625439683776831803,4713171619067320573891,4824287649379320273079,6839013035854228403323,6988663211693489418713,7000247134931341843087,7153425409218679738997,7172623427847117593729,7329573612263903536699,7341722604927992664701,7467543774379124667961,7502372990156176311631,7643595886938161005909,7831814202397618554203,7948042176803562738997,8016454222886363981807,8121959948724866081207,8135422346001289168993,8313440340443330507483,8425304899750395166457,8623936842377123539733,8678496818873036776279,8699385224423915536871,8836295382665048589211,8883097922657862790651,9044616688346739322159,9791570559169378166423,10022413087086927356987,10188256971222665177027,10428451634591372411063,10556330562351990315043,10807074756232085096873,11092899557954756777713,11219748183226937444081,11289690157300791142111,11356388858618765252243,11555851811303953212259,11840406750339854124653,12119551899660243612857,12432693932764984735333,13039166807534008380959,13780490424727643217919,17180935187633793305909,17556885629376327076279,17585986704827517313121,17970800418281073490651,18759927042952435141463,18878748848023683924413,19038333586296906095737,19202204301332453258747,19454927319038632706147,19487174456700762428053,19838339043479253822383,19913589652689838192343,19967032785628462490651,20403948163869785521081,20437768332637384985519,20788027263812157859459,20884984269894220543189,21166009870104651271831,21278118279854880638071,21665012067435212794939,21802077374242019218457,22316075269116094327733,23454227153359208166083,24007175534184965529527,24072972899627957654539,24598335794986486613209,24644777481566864012129,25178257267559841896821,31233353050604717749739,32148238864619339782937,51534775553466102497389,57106102640327302767377,59891766183757902902371,77006246582692837154477,80762648855019317015671,86905507114147634100763,88807159348592877735353,89493746028534918855203,94892446498817657366041,96300697072433864814359,98407933332224540193229,100998292051576980171157,103208320324040371422167,105151089363554701405613,107063020799267216627417,110280410795923223425399,118637401426215023830381,124424591739688927431863,143461672486675906952191,146767047495926058896243,147663944615454933579253,154227087402532263586913,187147950606785090153029,191593270098632716997519,241926141425870440875097,247219930078515308290307,249012629849678614928851,254461483894529350485281,264160053766978884018979,271897816838051704265857,298040301143906035476323,306770482725096411827009,334691978056117733842139,353706658032368794387513,370960641351020930699099,380545432141656836035231,389517033685271535509539,399108623953444974378413,408517864596748195778297,411064494470050220504407,442255502218682268905809,452681958066666919646221,596472113360858077620271,609524019561314490697301,625568314012607252138333,639256898564305441463023,651290119402775012543797,683060369129739647302031,693197320932889117234369,708365752463149272972539,734822319085696056236789,756904733359981771334683,770667310260608058980047,853982695153646768058971,956004378307089124673873,978542791941048003841037,1059356202988936597611589,1084331201880620761013041,1111032115329860334080447,1137225406850407139599043,1747929018820152585856739,1789137561503535358018391,1833193849006501492483897,1876412564503707814507093,2031376967818015167347021,2079267976882487037697049,2947614618453172441832213,3012113844239893939465303,3017106515155408334370497,3083126351373250967507707,3091400697402107682897199,3159046226885742424317269,3164282442723964838486131,3218511366757402731891191,3233522758757311990312961,3294389827270347393546779,3375511921233373596861493,3425606178202335540507707,3455091770064022876158817,3500564737900417281000217,3506367031126555631835983,3583092786731075448725173,3631306411792420316742967,3716916779064540245624923,3740432128934278850576249,3808443309928635941949941,3828615204665538862770581,3898229792677444647850529,4220166911002001989728313,4319660040534465690861397,4391138754596968691298637,4494662654508881509168153,4549778472373707825783533,4781039709478500171194303,4835711466970810038398911,4865856457796640982249841,4980572130672003834483629,5103215309396477127725443,5223526868753564997141367,5358491085021708420928523,5619880894047157612193329,5801586468810337794743899,5939391373057614226923089,7404983065870164914846779,7567017706261196969876249,7579560269780659961955151,7745414980279142674470581,8085528555512499545970553,8205521775693966527262647,8276150053874287354519957,8385073674505650696349357,8398972190838028606490843,8582757140309320260899833,8605791130605867333470581,8794101658627877559585911,8808678151366712928758689,8959639750703040037426829,9001428220324409054114459,9122550254015104698159161,9170868978617453555008601,9337620201064576714618709,9396695348298310283154967,9618228440989036655252923,10108771903097818719581773,10347092655233720143226137,10375451319739649749106309,10601882727639175730293079,10851828882318291857529851,13461575164810633350137509,22211488263543890176374659,24612730237981067492739487,25813351225199656150921901,37456273566197630297428853,38275885679243530303937143,40898644440990410324763671,41505600438218995734988729,42413819266188776823281699,43530263874229678453768667,44482786059661400082953977,45320119515692076305819203,46144161964484170366416727,47530857053042909296346969,51132720014698675270894211,53626999039805927723132953,61831980841757315896394321,80660766711524373855955499,104270166954550160017166807,106551789863840097873122317,113852983173567899012179949,128455369793023501290295213,2500483768057255589534620469.
**Primary headache associated with sexual activity:**
613582,3829598,167814677,1047395053.
**Primary stabbing:**
14937586453,15465416363,16204378237,16415510201,16521076183,16732208147,18632395823,19371357697.
**Nummular:**
331231589.
**Hypnic headache:**
14155130507,14655311797,15355565603,15555638119,15655674377,15855746893,17656399537,18356653343, 17956508311.
**Primary cough:**
1412526109,1440706181.
**Infrequent tension type headache:**
1378253359942681,1386197183342927,2206388426002661,2219105362175587,2241879928833803,2254801426982701,9699783080351321,9755689611073807,9855811762608983,9912617594093761,15777758744434123,15868696835180141,514088503258620013,517051549386911771,522358023418276099,525368732486969333,836221213455008519,841040932264547473,3676217787453150659,3697406362596972853,194839542735016984927,195962537217639561209.
**Frequent tension type headache:**
21262771073163,21385323067821,34038685022703,34234873409001,34586224191969,34785568423623,149641766231883,150504254798061,152048872391109,152925234768003,243408709879329,244811641924743,7931013610289799,7976725504297233,8058590236728777,8105037442704159,12900661623604437,12975017022011379,56714229401883657,57041112568465119,3005854158299833821,3023178966128651307.
**Chronic tension type headache:**
5029577936481697,5058566858305799,8051641932650957,8098049090764219,8181158961058211,8228312615012437,9281386088971379,9334881109657093,14858184597366199,14943822548936033,15097190247725977,15184205753476559,35396840949201377,35600857323548359,35966227130689871,36173525269771657,57576835707070051,57908690667917717,65319943607289139,65696427432115013,66370666560757597,66753206425661099,106250037026448857,106862429170693519,1876032570307672981,1886845438148063027,1906210037926563163,1917196839297897821,3051572292474712703,3069160605399639001,3461957011186324367,3481910653902095689,3517645327720152641,3537919940560038247,5631251962401789421,5663708746046756507,13415402719747321883,13492724925624828061,24756258627162583681,24898945996771589927,711016344146608059799,715114421058115887233,1312081707239616935093,1319644137828894266131.

We further generated, by hand, a list of mutually logically exclusive characteristics (Table 3). This list in Table 3 is then encoded as a list of composite numbers. We called this list L’.

**Table 3:** A list of logically contradicting characteristics.

1 min to 24 h with severe intensity	Constant
1 min to 24 h with severe intensity	Unremitting within 24 h
1 min to 24 h with severe intensity	Up to 72 h with mild intensity
1 min to 24 h with severe intensity	Up to few seconds
1 to 14 days per month	Constant
1 to 14 days per month	Every other day to 8 per day
1 to 14 days per month	Greater than 1 per day
1 to 14 days per month	Greater than 15 days per month
1 to 14 days per month	Greater than 5 per day
1 to 14 days per month	Less than 12 days per year
1 to 14 days per month	More than 1 episode per day
1 to 14 days per month	Unremitting within 24 h
1 to 600 s	15 min up to 4 h after waking
1 to 600 s	15 to 180 min
1 to 600 s	30 min to 7 days in duration
1 to 600 s	4 to 72 h
1 to 600 s	Greater than 5 min
1 to 600 s	Constant
1 to 600 s	Hours to days
1 to 600 s	Unremitting within 24 h
15 min up to 4 h after waking	4 to 72 h
15 min up to 4 h after waking	Constant
15 min up to 4 h after waking	Unremitting within 24 h
15 min up to 4 h after waking	Up to few seconds
15 to 180 min	4 to 72 h
15 to 180 min	Constant
15 to 180 min	Unremitting within 24 h
15 to 180 min	Up to few seconds
2 to 30 min	4 to 72 h
2 to 30 min	Constant
2 to 30 min	Hours to days
2 to 30 min	Unremitting within 24 h
2 to 30 min	Up to few seconds
30 min to 7 days in duration	Constant
30 min to 7 days in duration	Unremitting within 24 h
30 min to 7 days in duration	Up to few seconds
4 to 72 h	Between 1 s to 2 h
4 to 72 h	Constant
4 to 72 h	Unremitting within 24 h
4 to 72 h	Up to few seconds
Abrupt explosive intensity just before or with orgasm	Increasing in intensity with increasing sexual excitement
Aggravated by physical activity	Restless
Aggravated by physical activity	Not aggrevated by activity
Between 1 s to 2 h	Constant
Between 1 s to 2 h	Unremitting within 24 h
Bilateral location	Unilateral
Brought on by Primary exercise headache	Restless
Conjunctival injection	No conjunctival injection
Constant	Developing only during sleep and causing wakening
Constant	Each individual aura symptom lasts 5â€“60 min
Constant	Every other day to 8 per day
Constant	Fully reversible
Constant	Hours to days
Constant	Irregular frequency
Constant	Less than 12 days per year
Constant	Less than 48 h
Constant	Resolve within 1 h after removal of compression
Constant	Resolve within 1 h after removal of traction
Constant	Resolve within 30 min after removal of cold
Constant	Up to 72 h with mild intensity
Constant	Up to few seconds
Every other day to 8 per day	Unremitting within 24 h
Every other day to 8 per day	constant
Eyelid edema	NO eyelid edema
Forehead and facial sweating	No forehead and facial sweating
Fully reversible	Unremitting within 24 h
Greater than 15 days per month	Less than 12 days per year
Greater than 8 days per month	Less than 12 days per year
Hours to days	Resolve within 1 h after removal of compression
Hours to days	Resolve within 1 h after removal of traction
Hours to days	Resolve within 30 min after removal of cold
Hours to days	Unremitting within 24 h
Hours to days	Up to few seconds
Hours to days	Constant
Irregular frequency	Unremitting within 24 h
Irregular frequency	Constant
Lacrimation	No lacrimation
Less than 12 days per year	More than 10 days per month
Less than 12 days per year	Unremitting within 24 h
Less than 12 days per year	Constant
Less than 48 h	Unremitting within 24 h
Less than 48 h	Constant
Mild to moderate pain	Severe
Miosis	No miosis
Moderate to severe	Up to 72 h with mild intensity
More than 1 episode per day	Unremitting within 24 h
More than 1 episode per day	Constant
Nasal congestion	No nasal congestion
Nausea/vomiting	No nausea/vomiting
No orbital or supraorbital or temporal pain	Orbital or supraorbital or temporal pain
No phonophobia	Phonophobia
No photophobia	Photophobia
No ptosis	Ptosis
No restless	Restless
No rhinorrhea	Rhinorrhea
Nonpulsating	Pulsating
Severe	Up to 72 h with mild intensity
Unremitting within 24 h	Up to 72 h with mild intensity
Unremitting within 24 h	Up to few seconds

Any number in list M’ that was divisible by any number in list L’ was then excluded. The resulting list was therefore the totality of all possible patient clinical profiles with dual diagnosis that contains non-contradictory criteria. We then diagnosed these clinical profiles using the automated diagnosis technique discussed in Phase 1. Since multiple duplicates in dual diagnoses are possible – for example, there are multiple ways in which a patient profile may be able to satisfy concurrent diagnoses of cluster and migraine with aura—eliminating the duplicates generates the results presented in Table 4.

**Table 4:** Logically possible dual-diagnoses, classified.

Intersecting characteristics	
1. Dual diagnoses with intersecting characteristics with subset relationships:	
Migraine without aura	Chronic migraine
Migraine with aura	Chronic migraine
2. Dual diagnoses with intersecting characteristics without subset relationships:	
Migraine without aura	Migraine with aura
Cluster headache	Chronic tension type headache
Cluster headache	Infrequent tension type headache
Hemicrania continua	Chronic tension type headache
Hemicrania continua	Nummular headache
New daily persistent headache	Chronic tension type headache
Paroxysmal hemicrania	Infrequent tension type headache
Paroxysmal hemicrania	Short lasting unilateral neuralgiform headaches
Short lasting unilateral neuralgiform headaches	Primary headache associated with sexual activity
Short lasting unilateral neuralgiform headaches	Primary stabbing headache
New daily persistent headache	Hemicrania continua
New daily persistent headache	Nummular headache
New daily persistent headache	Primary thunderclap headache
Chronic tension type headaches	Chronic Migraine
**Non-intersecting characteristics**	
Migraine without aura	Cold stimulus headache
Migraine without aura	External compression headache
Migraine without aura	Primary exercise headache
Migraine without aura	Nummular headache
Migraine without aura	Primary headache associated with sexual activity
Migraine without aura	Primary thunderclap headache
Migraine without aura	External traction headache
Cluster headache	Chronic Migraine
Cluster headache	Primary Cough Headaches
Cluster headache	Hypnic headache
Cluster headache	Migraine with aura
Cluster headache	Migraine with aura
Cluster headache	Nummular headache
Cluster headache	paroxysmal hemicrania
Cluster headache	Primary headache associated with sexual activity
Cold stimulus headache	Cluster headache
Cold stimulus headache	External compression headache
Cold stimulus headache	frequent tension type headache
Cold stimulus headache	Hypnic headache
Cold stimulus headache	infrequent tension type headache
Cold stimulus headache	Migraine with aura
Cold stimulus headache	Nummular headache
Cold stimulus headache	paroxysmal hemicrania
Cold stimulus headache	Primary headache associated with sexual activity
Cold stimulus headache	Primary stabbing headache
Cold stimulus headache	Short lasting unilateral neuralgiform headaches
Cold stimulus headache	External traction headache
External compression headache	Cluster headache
External compression headache	Frequent tension type headache
External compression headache	Hypnic headache
External compression headache	Infrequent tension type headache
External compression headache	Migraine with aura
External compression headache	Nummular headache
External compression headache	Paroxysmal hemicrania
External compression headache	Primary headache associated with sexual activity
External compression headache	Primary stabbing headache
External compression headache	Short lasting unilateral neuralgiform headaches
External compression headache	External traction headache
Primary exercise headache	Chronic tension type headache
Primary exercise headache	Cluster headache
Primary exercise headache	Cold stimulus headache
Primary exercise headache	External compression headache
Primary exercise headache	Frequent tension type headache
Primary exercise headache	Hypnic headache
Primary exercise headache	Infrequent tension type headache
Primary exercise headache	Migraine with aura
Primary exercise headache	Nummular headache
Primary exercise headache	Paroxysmal hemicrania
Primary exercise headache	Primary headache associated with sexual activity
Primary exercise headache	Primary stabbing headache
Primary exercise headache	Short lasting unilateral neuralgiform headaches
Primary exercise headache	Primary thunderclap headache
Primary exercise headache	External traction headache
Hypnic headache	Chronic tension type headache
Hypnic headache	Frequent tension type headache
Migraine with aura	Chronic tension type headache
Migraine with aura	Frequent tension type headache
Migraine with aura	Hypnic headache
Migraine with aura	Infrequent tension type headache
Migraine with aura	Nummular headache
Migraine with aura	Primary headache associated with sexual activity
Migraine with aura	Primary stabbing headache
Nummular headache	Chronic tension type headache
Nummular headache	Frequent tension type headache
Nummular headache	Hypnic headache
Nummular headache	Infrequent tension type headache
paroxysmal hemicrania	Hypnic headache
paroxysmal hemicrania	Migraine with aura
Paroxysmal hemicrania	Nummular headache
Paroxysmal hemicrania	Primary headache associated with sexual activity
Primary headache associated with sexual activity	Chronic tension type headache
Primary headache associated with sexual activity	Frequent tension type headache
Primary headache associated with sexual activity	Hypnic headache
Primary headache associated with sexual activity	Infrequent tension type headache
Primary headache associated with sexual activity	Nummular headache
Primary headache associated with sexual activity	Primary stabbing headache
Primary stabbing headache	Nummular headache
Short lasting unilateral neuralgiform headaches	Migraine with aura
Short lasting unilateral neuralgiform headaches	Nummular headache
Primary thunderclap headache	chronic tension type headache
Primary thunderclap headache	Cluster headache
Primary thunderclap headache	Cold stimulus headache
Primary thunderclap headache	External compression headache
Primary thunderclap headache	frequent tension type headache
Primary thunderclap headache	Hemicrania continua
Primary thunderclap headache	Hypnic headache
Primary thunderclap headache	infrequent tension type headache
Primary thunderclap headache	Migraine with aura
Primary thunderclap headache	Nummular headache
Primary thunderclap headache	paroxysmal hemicrania
Primary thunderclap headache	Primary headache associated with sexual activity
Primary thunderclap headache	Primary stabbing headache
Primary thunderclap headache	External traction headache
External traction headache	Cluster headache
External traction headache	Frequent tension type headache
External traction headache	Hypnic headache
External traction headache	infrequent tension type headache
External traction headache	Migraine with aura
External traction headache	Nummular headache
External traction headache	Paroxysmal hemicrania
External traction headache	Primary headache associated with sexual activity
External traction headache	Primary stabbing headache
External traction headache	Short lasting unilateral neuralgiform headaches
Chronic migraine	Cold stimulus headache
Chronic migraine	External compression headache
Chronic migraine	Primary cough headache
Chronic migraine	Primary exercise headache
Chronic migraine	Hypnic headache
Chronic migraine	Nummular headache
Chronic migraine	paroxysmal hemicranias
Chronic migraine	Primary headache associated with sexual activity
Chronic migraine	Primary stabbing headache
Chronic migraine	Short lasting unilateral neuralgiform headaches
Chronic migraine	Primary thunderclap headache
Chronic migraine	External traction headache
Cold stimulus headache	Primary cough headache
Primary cough headache	External compression headache
Primary cough headache	Primary exercise headache
Primary cough headache	Frequent tension type headache
Primary cough headache	Hypnic headache
Primary cough headache	Infrequent tension type headache
Primary cough headache	Migraine with aura
Primary cough headache	Nummular headache
Primary cough headache	Paroxysmal hemicranias
Primary cough headache	Primary headache associated with sexual activity
Primary cough headache	Primary stabbing headache
Primary cough headache	Short lasting unilateral neuralgiform headaches
Primary cough headache	Primary thunderclap headache
Primary cough headache	External traction headache

### Analysis of results as “intersecting” vs. “non-intersecting” characteristics

We will now introduce a paradigm to interpret the results through the concept of “intersecting characteristics”: We defined a dual diagnosis pair as having “intersecting characteristics” if there exists at least one characteristic shared between the two diagnoses. If this does not exist then we described the pair as having “non-intersecting characteristics” [Non-intersecting characteristics are “less interesting” in the sense that it is always possible to obtain dual diagnosis whose characteristics are non-intersecting. We previously put forth this notion in a preprint (13)]. For example, primary stabbing headaches and SUNCT are a dual diagnosis pair with intersecting characteristics given that each contains duration measured in seconds. On the contrary, cold-induced headache and nummular headache are a dual diagnosis pair with non-intersecting characteristics as the characteristics in their criteria do not overlap.

We then classified our results through the following. First, we separated those with intersecting characteristics from those with non-intersecting characteristics. We further differentiated those with intersecting characteristics due to a subset relationship vs. those with intersecting characteristics without a subset relationship. For example, chronic migraine is a subset of migraine without aura since chronic migraine is defined as the existence of migraine without aura or migraine with aura greater than 15 days per month. This would be an example of an intersecting characteristic that contains a subset relationship.

### Software instrumentation

The above methods were implemented through custom code written by the author in Haskell Programming Language (Haskell 2010) via the Glasgow Haskell Compiler. The code is available for review upon inquiry to the author.

## Results

A total of 103 prime numbers were used to encode characteristics from the included ICHD3 diagnosis criteria (Table 1). A total of 578 encodings were generated (Table 2). A total of 99 pairs of illogical characteristics were found (Table 3). Once illogical characteristics were excluded, a total of 253,842 composite numbers representing unique dual-diagnosis clinical profiles were obtained. A number of profiles, although unique, yielded duplicate dual diagnoses; once these duplicates were removed, we obtained 145 possible logical dual diagnoses (Table 4).

Using our classification schema above, we obtained the following: 2 dual diagnoses with intersecting characteristics due to subset relationships and 14 dual diagnoses with intersecting characteristics without subset relationships. The remaining 129 dual diagnoses were the result of non-intersecting characteristics. This breakdown of results is shown in Table 4. The intersecting diagnosis that are subsets of each other contains only two pairs of dual diagnosis: migraine with or without aura and chronic migraine. This is to be expected given that we defined chronic migraine through the existence of migraine with aura or migraine without aura.

## Discussion

In clinical practice, patients with two concurrent ICHD3 diagnoses are common. Indeed, when managing secondary headaches with etiology which cannot be removed, it is recommended that one treat the phenotype of the disease; some would even suggest that secondary headaches may be triggers of underlying primary headaches and therefore should be treated according to the primary headache it provokes (14). Indeed, this is the preferred treatment method for post-traumatic headaches (15). Furthermore, medication overuse headache is by definition a co-diagnosis according to the ICHD3 (16).

Therefore, secondary headaches with a primary headache phenotype are a non-controversial manifestation of the dual diagnosis problem. In this way, the prevalence of co-diagnosis as a result of secondary headache is essentially the prevalence of secondary headaches in general – approximately 12.9% of headache patients seen at a tertiary headache center in one estimate to 18% of all patients with headaches by World Health Organization estimate in 2011 (17).

Dual diagnosis in primary headaches, however, can be a source of controversy as discussed in our introduction. This stems from the fact that primary headache disorders are phenotypically defined; controversies arise not necessarily from a disagreement in regards to pathophysiology, but rather toward an *a priori* definition. In other words, the problem is a Kantian one: What are the conditions of possibilities of being diagnosed with a specific kind of primary headache disorder? What are the redundancies in the contemporary classifications of headache disorders? And finally, how do our contemporary definitions of primary headaches inadvertently enable diagnostic uncertainties through the possibility of co-diagnosis? The results presented here may provide some answers. Our discussion will be organized around a few canonical diseases.

### The migraine/TTH dilemma

While the split between migraine without aura and tension-type headaches is an old one, that migraine with aura and tension-type headache can be co-diagnosed is not surprising; migraine with aura patients can often have a headache that is not phenotypically like migraine without aura—the exemplary case being migraine with aura without headaches. However, this study demonstrates that it is possible to have a migraine with aura diagnosed concurrently with a chronic tension-type headache and chronic migraine. This is due to the “loophole” that allows for the co-existence of migraine with aura and chronic tension-type headache. In this case, if aura occurs greater than 15 days per month, one can obtain diagnoses that are a combination of chronic migraine, migraine with aura, and chronic tension-type headache.

### Cluster headaches, TAC, and SUNCT/SUNA

Our results highlight some of the peculiarities of our contemporary classification of TAC. We show that cluster headache can be diagnosed together with hypnic headache and that cluster headache has a non-intersecting phenotypical relationship with migraine with aura. This is, of course, a source of contemporary debate mentioned previously. Indeed, one can make the same argument for any TAC being co-diagnosed with migraine with aura following the same logic. If we use de Coo et al.’s estimate, an aura is prevalent in 7.0% of cluster headaches diagnoses; we can therefore estimate that migraine with aura can be co-diagnosed in approximately 7% of cluster headache patients (4). Our study demonstrates that since cluster headache can be diagnosed together with migraine with aura, it is theoretically possible to have a chronic migraine form of cluster headache with migraine with aura. If a patient contains a co-diagnosis of migraine with aura with 15 days of aura and cluster headache, that person satisfies a chronic migraine diagnosis.

Our data also support the dilemma between paroxysmal hemicranias and cluster headaches in clinical diagnosis. Fortunately, an indomethacin trial can often be used to differentiate the two (16). Nevertheless, a co-diagnosis between cluster headache and paroxysmal hemicranias is possible as cluster headache is not defined as unresponsive to an indomethacin trial.

Short-lasting unilateral neuralgia form headaches can be co-diagnosed with sex headaches and primary stabbing headaches. This is due to the fact that all of these headache types are short lasting in nature and raises the theoretical possibility of subtypes of short-lasting unilateral neuralgia form headaches to be further classified by their provoking characteristics.

### New daily persistent headaches and status migrainosus

New daily persistent headaches (NDPH) can be logically diagnosed with chronic tension-type headaches; empirically, multiple researchers of new daily persistent headaches separate migraine vs. tension-type headaches characteristics in NDPH (8). Specifically, Lobo et al. studied NDPH’s primary headache phenotypes; if we interpret this to mean co-diagnoses of a primary headache disorder with NPDH then the prevalence of co-diagnosis of TTH in NDPH was 8.8% whereas the prevalence of co-diagnosis with chronic migraine was 89.7% according to Lobo et al.’s study (7). Even though it may be intuitively obvious what kind of clinical phenomenology a migraine-like NDPH represents, according to the ICHD3 definition, it is actually impossible to diagnose a new daily persistent headache concurrently with either migraine with or without aura. This is due to (1) a limitation of the current study of not including status migrainosus and (2) from an artifact of the classification guideline which suggests that a migraine, both migraine without aura as well as migraine with aura, must not be constant. Indeed, migraine without aura is limited to a 72-h duration according to the classification, and migraine with aura also is limited by the fact that the migraine aura must be reversible. It is only the concept of status migrainosus which allows one to bridge this gap and therefore allows for the possibility of migrainous NDPH.

The concurrent diagnosis of new daily persistent headaches with hemicranias continua, nummular headaches, or thunderclap headaches coincidentally describes the sub-classification of new daily persistent headaches that was presented by Rosen in a prior review article (18). For instance, the lock-sided headache nature of some new daily persistent headaches may be attributed to a cervicogenic cause, of which nummular headache, being lock sided in nature, can be another manifestation. That new daily persistent headache can be concurrently diagnosed with thunderclap headache coincides with Rosen’s proposal of a persistent RCVS subtype of NDPH (18, 19).

### Non-intersecting characteristics

Non-intersecting characteristics represent the majority of dual diagnoses. This leads to the question of headache triggers in primary headache disorders. For example, it is possible to co-diagnose migraine without aura with cold-induced headaches, compression headaches, or exercise headaches. These potential co-diagnoses invite the question of whether stimulus-related headaches and migraine can be interpreted as the same entity.

### Secondary headaches: post-traumatic headaches and medication overuse headaches

While this project is limited to primary headache disorders, some of the implications of our methodology and results can be translated to secondary headaches. We can examine two such cases: post-traumatic headaches (PTH) and medication overuse headaches (MOH).

Post-traumatic headaches represent approximately 4% of symptomatic headache disorders (20). According to ICHD3, PTH is characterized by combinations of the following: a temporal relationship with the insult, regain of consciousness following the trauma, discontinuation of medication which can hinder the ability to report the existence of headache, and whether there is a resolution of headache within 3 months (see ICHD3 criteria 5.1 and 5.2). Notice that none of these are among the list of characteristics of primary headaches (see Table 1). In the language of this article then, PTH forms a “non-intersecting characteristic” with each of the primary headache disorders. Therefore, PTH can be concurrently diagnosed, at least theoretically, with any of the primary headache disorders. Even though this statement is a theoretical one, we do have some empirical evidence: It is well established that PTH often exhibits migraine or tension-type headache phenotypes (15). Furthermore, we have also observed cases of post-traumatic trigeminal autonomic cephalalgias (21–24). Of note, sub-classifications of acute or posttraumatic headaches do involve nausea or vomiting in 5.1.2 and 5.2.2. Yet, given the fact that these criteria are listed under a logical OR statement, PTH can easily satisfy tension-type headache criteria or migraine criteria as a concurrent diagnosis.

Medication overuse headache has a prevalence of approximately 30 to 50% of patients seen in headache centers (25). Medication overuse headache is unique in that it must be part of a dual diagnosis. This is due to its definitional requirements: (1) An existence already of another headache disorder, (2) occurrence of more than 15 days/month, and (3) regular overuse for more than 3 months of the inciting medication (see 8.2 of ICHD3). “Regular overuse for more than 3 months of medication” is not a characteristic that is within the criteria for any of the primary headache disorders. However, “the occurrence of more than 15 days/month” is a characteristic that is found in primary headache disorders; specifically, this criterion is logically contradictory to two characteristics according to Table 3: “1 to 14 days per month” and “less than 12 days per year.” Therefore, primary headache disorders containing any of these two characteristics cannot be concurrently diagnosed with MOH. Since “1 to 14 days/month” and “no more than 12 days per year” arise from criteria of frequent and infrequent episodic tension-type headaches, respectively, we can therefore deduce that these headaches cannot be concurrently diagnosed with MOH. For the same reason, episodic migraine (i.e., those with less than 15 days per month) cannot carry a concurrent diagnosis with MOH; in other words, MOH diagnosis in migraine implies a concurrent diagnosis with chronic migraine.

### Diagnostic uncertainty and co-diagnoses

Our approach in this article can be described as meta-scientific in nature (26). Highlighting *a priori* co-diagnoses in the ICHD3 leads to a fundamental question: Are co-diagnoses an inevitable by-product of headache taxonomy or should we eliminate them in future classifications? In other words, are co-diagnoses simply the loci of diagnostic uncertainties or are they inevitable by-products of shared pathophysiology between different disorders? For example, when a patient receives a dual diagnosis of both cluster headache and migraine with aura, should that reflect a diagnostic uncertainty between cluster headaches vs. migraine with aura or alternatively an appropriate description of that particular patient’s underlying pathophysiology?

If it is the former, then future revisions and constructions of ICHD may benefit from our meta-scientific project. For example, clarifying, amending, or even adding new characteristics to our current criteria of cluster headaches and/or migraine with aura, may prevent a co-diagnosis of migraine with aura and cluster headache, and as a result, significantly decrease clinical diagnostic uncertainty. If so, our project offers a comprehensive list of co-diagnoses for this kind of future work.

### Limitations

First, although phase one of this study constructs an algorithm of automated diagnosis, it is not intended to be a substitute for detailed clinical history taking. Indeed, it is only through careful communication with our patients that accurate input data can be obtained in the first place.

Second, despite our attempt to rule out obvious logical contradictions, this is not a foolproof method. For example, concurrent diagnosis of cold-induced headache with hypnic headache is on paper logically consistent but in practice impossible—it would be unlikely for one to be woken up at night from a hypnic headache while triggering that same headache through a cold stimulus. However, these sorts of occurrences in the data appear to be rare. The only other two instances that follow a similar argument are the compression headache and hypnic headache pairing as well as the primary cough headache and hypnic headache pairing.

This study is also limited by having included only four headache categories—migraine, tension-type headache, trigeminal autonomic cephalalgia, and other primary headaches. Other primary headaches, such as those described in the addendum section of the ICHD3, have been excluded. The future direction for our project will involve the inclusion of other primary headaches as well as secondary headaches.

Status migrainosus is considered a “complication of migraine” in the ICHD3 and therefore excluded based on our inclusion criteria. This proves to be an important omission as it limits the duration of migraine attacks. The complicated relationship of status migrainosus to intractable headaches is not lost for those conducting research on status migrainosus (27). As such, although our omission causes the study to be incomplete, it does show us the importance of rethinking duration in migraine definitions.

Finally, even though the clinical utility of our result may be limited in clinical practice, our project may carry implications for machine learning algorithms in headaches as well as clinical trial design. In most machine learning diagnostic classification algorithms, the goal of classification is binary or, alternatively, organized in sequential layers of binary classification (28, 29). Our study on concurrent diagnosis challenges this *principium tertii exclusi* approach. This project’s identification of the specific cases in which this assumption is violated will likely improve the data science endeavor to construct automated diagnostic algorithms in the future. Indeed, this same concern can be carried over into clinical trial design: the inclusion of co-diagnosed patients in clinical trials—such as those co-diagnosed with migraine with aura/cluster in a cluster trial—may not be methodologically sound. Our article, therefore, stresses that inclusion/exclusion criteria in clinical trials must take into account the possibility of co-diagnoses in trial design.

## Conclusion

Prime number encoding of ICHD3 allows for an exhaustive study of the structure of contemporary headache diagnoses. In our pilot study of this approach, these important results are obtained: (1) Status migrainosus is required for modeling migraine intractability in our classification and (2) the possibility of a dual diagnosis of chronic migraine and migraine with aura allows for loopholes in diagnoses which enable the construction of large concurrent diagnoses in headache diagnoses such as co-diagnoses of chronic migraine, chronic tension-type headaches, and migraine with aura. This may be undesirable in our classification.

## Data availability statement

The original contributions presented in the study are included in the article/supplementary material, further inquiries can be directed to the corresponding author.

## Author contributions

PZ was responsible for the entirety of the manuscript, including conceptualization, data analysis, as well as drafting of manuscript.

## Funding

Article processing fee is supported by Rutgers Neurology Department.

## Conflict of interest

PZ has received an honorarium from Lundbeck Biopharmaceuticals, Board Vitals, and Fieve Clinical Research, collaborates with Headache Science Incorporated without receiving financial support, and has ownership interest in Cymbeline LLC.

## Publisher’s note

All claims expressed in this article are solely those of the authors and do not necessarily represent those of their affiliated organizations, or those of the publisher, the editors and the reviewers. Any product that may be evaluated in this article, or claim that may be made by its manufacturer, is not guaranteed or endorsed by the publisher.

## Abbreviations

ICHD3: International classification of headache disorders, 3rd edition
NDPH: New daily persistent headache
