# Corrigendum: Which headache disorders can be diagnosed concurrently? An analysis of ICHD3 criteria using prime encoding system

**DOI:** 10.3389/fneur.2024.1404283

**Published:** 2024-04-08

**Authors:** Pengfei Zhang

**Affiliations:** Department of Neurology, Rutgers Robert University Medical School, New Brunswick, NJ, United States

**Keywords:** headaches classification, International Classification of Headache Disorders, 3rd edition, headache diagnosis, number theory in medicine, automated diagnosis

In the published article, there was an error in the legend for Table 4 as published. The error is that Table 4 contains two titles that state “Non-intersecting characteristics”. The first one of the two should say “Intersecting characteristics”. The corrected legend appears below.

“Intersecting characteristics”

In the published article, there was an error in Table 2 as published. The encoding (number portion) for hypnic headache appears to be missing one value in Table 2. One more number, 17956508311, should be added to the existing list for hypnic headache. The corrected Table 2 and its caption appears below.

**Table 2:** Prime number encodings for selected primary headache disorders.

**Migraine without aura:**
7580756461,13242732241,16451185183,102951563551,127894697713,223417708453,3191498470081,3964735629103,4166259653173,6925948962043,7277988854713,9041302068919,53843667737173,56580493999543,70288832213209,122786715194029,1669153699852363,1753995313985833,2178953798609479,3806388171014899,29591598361760989,917339549214590659.
**New Daily Persistent Headache:**
1145520433.
**Primary exercise headache:**
2106413.
**Primary thunderclap headache:**
17411171.
**Cold-stimulus headache**
4026427.
**External compression headache:**
1069111207.
**External traction headache:**
1183350401.
**Cluster headache:**
1577982193416431,1932584933510011,2251727399594233,3492836989921763,4237502744118281,4911247950296083,7428927404960501,7960831515100871,8173593159157019.
**Paroxysmal Hemicrania:**
699200435231955421,856324128542507201,997735452522003803,1547668379108935033,1877628135061093771,2176163152351142153,3291741374856059791,3527426914821887461,3621701130808218529.
**Short lasting unilateral neuralgiform headaches:**
444401003390335,544266397410635,634145252028905,983674131099955,1193391458542585,1383135707181155,2092180004725285,2301897332167915.
**Hemicrania continua:**
54659995058027,66943140014887,77997970476061,120988977825071,146783582234477,170121557652511,257331886846217,275756604281507,283126491255623.
**Migraine with aura:**
75121389451,179941932871,188719588133,209121705769,345049093919,581872457273,594604896163,635348700611,716836309507,826512945899,866830650577,889227610013,910191718697,932604566599,954591314731,960542072261,1033426681907,1057790375783,1393787513933,1424286146623,1461777148759,1493763519629,1521881771231,1596119906413,1619807110787,1655251467697,1717073020447,1768673409809,1800832679981,1995517294033,2233913264179,2286579195751,2475417400847,2533776946643,2596169469181,2657375822089,4084418344297,4180711114693,4283658263531,4384648242239,4746756454183,4858664268427,6887746063999,7038462608069,7050129075331,7204398858161,7223733676877,7381802247487,7394037810713,7520755549093,7555832948803,7698062163217,7887621673439,8004677858161,8073577390691,8179834922891,8193393249709,8372679754079,8485341431141,8685388773329,8740337530027,8899260525343,8946396568063,9109066274467,9861342744299,10093830196031,10260855840551,10502762068619,11299697190653,11370137553043,11638195805767,11924778409289,12205912674341,12521286076129,13132080518867,17303362063217,17681991430027,17711299872173,18098855668063,18893605403819,19173995799781,19339034214911,19593558071111,19626034993489,20055488713259,20109312668063,20549341391653,20583402554147,20936157339367,21033805235857,21316833351403,21429740616523,21819391308607,21957433307141,22475093817329,23621355875879,24178244423051,24773617138117,25357670980273,31455913801007,51901998960457,57513025875101,60318539332423,87524773154719,89439976068389,95568625392133,96986910793067,99109162670377,101717979612241,103943755971371,105900368677769,107825924039621,111066240320587,119482780692553,125311209019019,144483943052083,243650044187461,248981555001191,266042389605127,300164058812999.
**Chronic migraine:**
47943440512446421,83751819318423001,104043233975143063,651102853410965911,808852238322886393,1412974242049523533,20184188455739943241,20663622860864407451,25074419388009478183,26348930066400847453,36097034126240313431,43802201503535229523,44842633843286660153,46028628871181563393,57180458193890635759,74589881356425382327,178668785574693357667,187384336090532058041,207642102154373361613,280625329820126307641,340526792333935171453,342607760128665739163,348615314717164035383,357835469611443767023,444531949184762684449,577755521692989825821,590397874465265314351,608991898323344642723,630853403336546877647,711764461079110004239,776547512891224440469,820665099843083049623,860697543737867588629,882936037451639990801,903751818295293585869,926006088059037063523,947837272846283516887,953745926844664084697,1026114854335689719039,1050306167208043897091,1383926017078557024641,1414208862091216915771,11451434603277511025843,1483194660242007984833,1511113966131728567387,1584826842528398253601,1608346452280485190799,1643540028916819658869,1704924174212751870619,1756159474153089956693,1788091207101178791137,1981398364625630552341,2218107606280949245183,2270400909375981447427,2457903023176187001419,2515849656335547009311,2577800731623805879537,2638574029815329790253,4055519765243973517069,4151131233186856979161,4253349997694899054487,4353625439683776831803,4713171619067320573891,4824287649379320273079,6839013035854228403323,6988663211693489418713,7000247134931341843087,7153425409218679738997,7172623427847117593729,7329573612263903536699,7341722604927992664701,7467543774379124667961,7502372990156176311631,7643595886938161005909,7831814202397618554203,7948042176803562738997,8016454222886363981807,8121959948724866081207,8135422346001289168993,8313440340443330507483,8425304899750395166457,8623936842377123539733,8678496818873036776279,8699385224423915536871,8836295382665048589211,8883097922657862790651,9044616688346739322159,9791570559169378166423,10022413087086927356987,10188256971222665177027,10428451634591372411063,10556330562351990315043,10807074756232085096873,11092899557954756777713,11219748183226937444081,11289690157300791142111,11356388858618765252243,11555851811303953212259,11840406750339854124653,12119551899660243612857,12432693932764984735333,13039166807534008380959,13780490424727643217919,17180935187633793305909,17556885629376327076279,17585986704827517313121,17970800418281073490651,18759927042952435141463,18878748848023683924413,19038333586296906095737,19202204301332453258747,19454927 319038632706147,19487174456700762428053,19838339043479253822383,19913589652689838192343,19967032785628462490651,20403948163869785521081,20437768332637384985519,20788027263812157859459,20884984269894220543189,21166009870104651271831,21278118279854880638071,21665012067435212794939,21802077374242019218457,22316075269116094327733,23454227153359208166083,24007175534184965529527,24072972899627957654539,24598335794986486613209,24644777481566864012129,25178257267559841896821,31233353050604717749739,32148238864619339782937,51534775553466102497389,57106102640327302767377,59891766183757902902371,77006246582692837154477,80762648855019317015671,86905507114147634100763,88807159348592877735353,89493746028534918855203,94892446498817657366041,96300697072433864814359,98407933332224540193229,100998292051576980171157,103208320324040371422167,105151089363554701405613,107063020799267216627417,110280410795923223425399,118637401426215023830381,124424591739688927431863,143461672486675906952191,146767047495926058896243,147663944615454933579253,154227087402532263586913,187147950606785090153029,191593270098632716997519,241926141425870440875097,247219930078515308290307,249012629849678614928851,254461483894529350485281,264160053766978884018979,271897816838051704265857,298040301143906035476323,306770482725096411827009,334691978056117733842139,353706658032368794387513,370960641351020930699099,380545432141656836035231,389517033685271535509539,399108623953444974378413,408517864596748195778297,411064494470050220504407,442255502218682268905809,452681958066666919646221,596472113360858077620271,609524019561314490697301,625568314012607252138333,639256898564305441463023,651290119402775012543797,683060369129739647302031,693197320932889117234369,708365752463149272972539,734822319085696056236789,756904733359981771334683,770667310260608058980047,853982695153646768058971,956004378307089124673873,978542791941048003841037,1059356202988936597611589,1084331201880620761013041,1111032115329860334080447,1137225406850407139599043,1747929018820152585856739,1789137561503535358018391,1833193849006501492483897,1876412564503707814507093,2031376967818015167347021,2079267976882487037697049,2947614618453172441832213,3012113844239893939465303,3017106515155408334370497,3083126351373250967507707,3091400697402107682897199,3159046226885742424317269,3164282442723964838486131,3218511366757402731891191,3233522758757311990312961,3294389827270347393546779,3375511921233373596861493,3425606178202335540507707,3455091770064022876158817,3500564737900417281000217,3506367031126555631835983,3583092786731075448725173,3631306411792420316742967,3716916779064540245624923,3740432128934278850576249,3808443309928635941949941,3828615204665538862770581,3898229792677444647850529,4220166911002001989728313,4319660040534465690861397,4391138754596968691298637,4494662654508881509168153,4549778472373707825783533,4781039709478500171194303,4835711466970810038398911,4865856457796640982249841,4980572130672003834483629,5103215309396477127725443,5223526868753564997141367,5358491085021708420928523,5619880894047157612193329,5801586468810337794743899,5939391373057614226923089,7404983065870164914846779,7567017706261196969876249,7579560269780659961955151,7745414980279142674470581,8085528555512499545970553,8205521775693966527262647,8276150053874287354519957,8385073674505650696349357,8398972190838028606490843,8582757140309320260899833,8605791130605867333470581,8794101658627877559585911,8808678151366712928758689,8959639750703040037426829,9001428220324409054114459,9122550254015104698159161,9170868978617453555008601,9337620201064576714618709,9396695348298310283154967,9618228440989036655252923,10108771903097818719581773,10347092655233720143226137,10375451319739649749106309,10601882727639175730293079,10851828882318291857510851828882318291857529851,13461575164810633350137509,22211488263543890176374659,24612730237981067492739487,25813351225199656150921901,37456273566197630297428853,38275885679243530303937143,40898644440990410324763671,41505600438218995734988729,42413819266188776823281699,43530263874229678453768667,44482786059661400082953977,45320119515692076305819203,46144161964484170366416727,47530857053042909296346969,51132720014698675270894211,53626999039805927723132953,61831980841757315896394321,80660766711524373855955499,104270166954550160017166807,106551789863840097873122317,113852983173567899012179949,128455369793023501290295213,2500483768057255589534620469.
**Primary headache associated with sexual activity:**
613582,3829598,167814677,1047395053.
**Primary stabbing:**
14937586453,15465416363,16204378237,16415510201,16521076183,16732208147,18632395823,19371357697.
**Nummular:**
331231589.
**Hypnic headache:**
14155130507,14655311797,15355565603,15555638119,15655674377,15855746893,17656399537,18356653343, 17956508311.
**Primary cough:**
1412526109,1440706181.
**Infrequent tension type headache:**
1378253359942681,1386197183342927,2206388426002661,2219105362175587,2241879928833803,2254801426982701,9699783080351321,9755689611073807,9855811762608983,9912617594093761,15777758744434123,15868696835180141,514088503258620013,517051549386911771,522358023418276099,525368732486969333,836221213455008519,841040932264547473,3676217787453150659,3697406362596972853,194839542735016984927,195962537217639561209.
**Frequent tension type headache:**
21262771073163,21385323067821,34038685022703,34234873409001,34586224191969,34785568423623,149641766231883,150504254798061,152048872391109,152925234768003,243408709879329,244811641924743,7931013610289799,7976725504297233,8058590236728777,8105037442704159,12900661623604437,12975017022011379,56714229401883657,57041112568465119,3005854158299833821,3023178966128651307.
**Chronic tension type headache:**
5029577936481697,5058566858305799,8051641932650957,8098049090764219,8181158961058211,8228312615012437,9281386088971379,9334881109657093,14858184597366199,14943822548936033,15097190247725977,15184205753476559,35396840949201377,35600857323548359,35966227130689871,36173525269771657,57576835707070051,57908690667917717,65319943607289139,65696427432115013,66370666560757597,66753206425661099,106250037026448857,106862429170693519,1876032570307672981,1886845438148063027,1906210037926563163,1917196839297897821,3051572292474712703,3069160605399639001,3461957011186324367,3481910653902095689,3517645327720152641,3537919940560038247,5631251962401789421,5663708746046756507,13415402719747321883,13492724925624828061,24756258627162583681,24898945996771589927,711016344146608059799,715114421058115887233,1312081707239616935093,1319644137828894266131.

“Prime number encodings for selected primary headache disorders.”

The authors apologize for this error and state that this does not change the scientific conclusions of the article in any way. The original article has been updated.

