# Supplementary material for: Which headache disorders can be diagnosed concurrently? An analysis of ICHD3 criteria using prime encoding system
Source: Front Neurol. 2023 Aug 21;14:1221209. doi: 10.3389/fneur.2023.1221209 (PMC10475541; doi:10.3389/fneur.2023.1221209)
Supplement: Supplementary file 1 [file Presentation_1.pdf]

SUPPLEMENTAL MATERIAL

PENGFEI ZHANG*

1. Mathematical Justification of the Technique. This supplemental material is intended to establish a formal mathematical treatment of Phase 1 of the study and is excerpted and modified from a prior work by the author as cited in reference 10 of the main manuscript.

We begin our construction with a formal definition of "encoding" for a given collection of diagnostic criteria. We will first develop the following abstraction:

DEFINITION 1.1. *Let S be the following sequence:*

$$S = \{S_1, S_2, S_3...S_i...S_{m-1}, S_m\}$$

where S_i represents individual statements (i.e. phenotype) in a classification.

We now define a function that "encodes" the propositional values for each individual statements based on whether the users answers $True$ or $False$.

DEFINITION 1.2. *Define the following:*

$$\varphi(S_i) = \begin{cases} P_i^1 & \text{if } S_i = True \\ P_i^0 & \text{if } S_i = False \end{cases}$$

where P_i is the ith prime. Here "True" or "False" assigned by the user for each S_i statement.

DEFINITION 1.3. *The "encoding" for a set of user assigned phenotype is the following:*

$$Q(S) = \prod_i^m \varphi(S_i))$$

Notice that the "space" in which these encodings exist is simply the square free integers up to the mth prime. We will call this I_m, representing all possible values of a user's questionnnaire:

DEFINITION 1.4.

$$I_m = \{i | i \in P_1^{n_1} P_2^{n_2} P_3^{n_3} ... P_m^{n_m}, n_m \leq 1, m \in \mathbb{Z}, p \in \mathbb{P}\}$$

We will first prove that prime factorization and propositional values in the user's answers are connected for logic conjunction:

THEOREM 1.5. *Given $a \in I_m$, if P_i divides a, then $S_i = True$.*

Proof. By fundamental theorem of arithmetic:

$$a = P_1^{n_1} P_2^{n_2} P_3^{n_3} ... P_m^{n_m}$$

Since P_i divides a, then $n_i \geq 1$. Now I_m is square free and $a \in I_m$, therefore $n_i = 1$. Suppose to the contrary that $S_i = False$, then $\varphi(S_i) = P_i^0$. This implies that $n_i = 0$, reaching a contradiction. ∎

*Rutgers Robert Wood Johnson Hospital, New Brunswick, NJ (pz124@rwjms.rutgers.edu).

Now we will prove the following relationship between two encoding:

THEOREM 1.6. *Let $\iota, \pi \in I_m$, where $\pi = P'_{a1}P'_{a2}...P'_{aj}$ and the user assigns $S_1, S_2...S_j$ such that ι is the encoding by definition 4.3. If π divides ι, then $S_{a1} = True, S_{a2} = True...S_{aj} = True$*

(We use the subscript $a1, a2, ...aj$ to differentiate arbitrary collections of prime from the jth prime.)

Proof. Given that π divides ι, then P'_{a1} divides ι, P'_{a2} divides ι, P'_{a3} divides ι....P'_{aj} divides ι
By Theorem 4.5, $S_{a1} = True, S_{a2} = True...S_{aj} = True$ □

We now need to define the notion of diagnostic criteria and what it means to diagnose a disorder.

DEFINITION 1.7. *A diagnostic statement, σ, for a specific disorder is the assignments of $True$ to a subsequence of S and the assignments of $False$ otherwise.*

DEFINITION 1.8. *A diagnostic criteria for a specific disorder is a collection of diagnostic statements for that disorder.*

For example, a migraine diagnostic statement is assigning each of S_1, S_2, S_3, S_4, S_5 to $True$ if S_1 is "at least five headaches", S_2 is "each lasting 4 to 72 hours", S_3 is "unilateral", S_4 is "pulsating", and S_5 is "nausea". This diagnostic statement describes one of a number of conditions that satisfies migraine without aura according to ICHD3. A diagnostic criteria is the collection of all such statements which satisfies migraine without aura. We are able to define a diagnostic criteria as a collection of diagnostic statements due to the fact that all propositional statements can be translated into a disjunctive normal form.

DEFINITION 1.9. *We say that i', an assignment of $True$ and $False$ to elements of S, is considered a "diagnosis" of a diagnostic criteria, $M' = [\sigma_1, \sigma_2, ...\sigma_j..., \sigma_k]$, if σ_j^T is a subsequence of i'^T for some j, where σ_j^T the subsequent of σ_j that is assigned as $True$ and i'^T is the subsequent of i' that is assigned as $True$.*

This should be intuitive as any assignment that conforms to a diagnostic criteria is the diagnosis. The i' here is really just the representation for a set of phenotype, such as a patient profile. (This set needs to be ordered in order to be assigned a prime number by φ, therefore sequence is used and not set in our definitions.) The definition simply suggests that if there is a diagnostic statement, σ_j, which matches a subsequence of $True$ as i', then it is diagnostic. Notice that $False$ is not taken in to consideration here, specifically due to the fact that negations are assigned a prime number and encoded directly.

We can pin down these definitions mathematically by directly considering encodings:

DEFINITION 1.10. *A diagnosis set, M, for a diagnostic criteria*

$$M' = [\sigma_1, \sigma_2, ...\sigma_k]$$

is defined as the following:

$$M = [Q_1(S), Q_2(S), Q_3(S)...Q_n(S)]$$

where σ_1 assigns $True$ or $False$ to elements of S in $Q_1(S)$ in accordance to Definition 4.3. The same applies to σ_2 to $Q_2(S)$ ... etc.

This is the mathematical encoding for a diagnostic criteria where every element in the set M is a composite number encoding a diagnostic statement. A concrete example of this is shown in section 3.

Now we find prove the crux of our argument:

THEOREM 1.11. *Let M be a diagnostic set for diagnostic criteria M',i an encoding for i', and let $\pi \in M$ where π encodes $\sigma \in M'$. Then if π divides i, then i' is diagnostic of M'.*

Proof. Since $\pi \in M$, $\pi \in I_m$. Similarly, since i is an encoding, $i \in I_m$ also. Let $\pi = P'_{a1}P'_{a2}...P'_{aj}$. By theorem 4.6, since π divides i, then $S_{a1} = True, S_{a2} = True...S_{aj} = True$ for i'. Furthermore, $S_{a1} = True, S_{a2} = True...S_{aj} = True$ for σ by theorem 4.6, since π divides itself. Since $\sigma \in M$, and the above suggests that whichever element of S is $True$ for σ is $True$ also for i', therefore the former is a subsequence of the latter. By definition 4.9 i' is diagnostic of M'. □

THEOREM 1.12. *Let i' be diagnostic of M'. Then there exists $\pi \in M$ where π encodes $\sigma \in M'$ such that π divides i.*

Proof. Let i be an encoding of i' and i' be a diagnostic of M'. Then there exists σ_j such that σ_j^T is a subsequence of i'^T by definition 4.9. Applying definition 4.3 to this σ_j yield a π such that $\pi = Q_j(S)$. Therefore $\pi \in M$ by definition 4.10. Now we need to show that this π divides i: Since σ_j^T is a subsequence of i'^T, so $Q(\sigma_j)$ divides $Q(i')$. □

The above two theorems therefore justify the following claim:

THEOREM 1.13. *Let M be the diagnostic set of M', i be an encoding of i', and $\pi \in M$ where π encodes σ. Then π divides i if and only if i' is diagnostic of M'.*
